# The effectiveness of dual-phase ^18^F-FDG PET/CT in the detection of epithelial ovarian carcinoma: a pilot study

**DOI:** 10.1186/1757-2215-7-15

**Published:** 2014-02-05

**Authors:** Jae Kwan Lee, Kyung-Jin Min, Kyeong A So, Sungeun Kim, Jin Hwa Hong

**Affiliations:** 1 Department of Obstetrics and Gynecology, Guro Hospital, College of Medicine, Korea University, Seoul, Republic of Korea; 2 Department of Nuclear Medicine, Guro Hospital, College of Medicine, Korea University, Seoul, Seoul, Republic of Korea

**Keywords:** Standardized uptake value, Ovarian cancer, Positron emission tomography, Retention index, Dual-phase

## Abstract

**Background:**

The aim of our study is to establish the potential role of dual-phase ^18^F-fluorodeoxyglucose positron emission tomography / computed tomography (FDG-PET/CT) in patients presenting ovarian masses with diffuse peritoneal infiltration for differentiating benign from malignant lesions.

**Methods:**

Twenty patients (13 with ovarian cancers and 7 with benign lesions) were evaluated preoperatively by dual-phase ^18^F-FDG-PET/CT performed 1 h and 2 h after injection of ^18^F-FDG. The maximum standardized uptake value (SUV_max_) for both time points SUV_max1_ and SUV_max2_ were determined, respectively, and the retention index (RI) was calculated by subtracting the SUV_max1_ from the SUV_max2_ and dividing by SUV_max1_.

**Results:**

The areas under the receiver operating characteristic curves (AUCs) of SUV_max1_ and SUV_max2_ were 0.753 (P = 0.062, 95% confidence interval [CI] = 0.512–0.915) and 0.835 (P = 0.001, 95% CI = 0.604–0.961), respectively. The AUC of the RI was 0.901 (P < 0.001, 95% CI = 0.684–0.988). Using pairwise comparisons, the AUC of SUV_max2_ was significantly higher than that of SUV_max1_ (P = 0.032). The AUC of the RI was higher than those of SUV_max1_ and SUV_max2_, but the difference was not statistically significant.

**Conclusion:**

Dual-phase ^18^F-FDG PET/CT might be considered when preoperative imaging is indeterminate. A larger-scaled, prospective study is needed to verify these results.

## Background

Epithelial ovarian cancer is the leading cause of death among all other gynecological malignancies. The symptoms of this cancer are usually nonspecific, and most women are diagnosed at advanced stages. Therefore, early detection and characterization is crucial in patients who present with an adnexal mass for prompt surgical intervention and for improving survival. However, characterization of adnexal masses is often challenging to clinicians because both benign and malignant adnexal masses have overlapping imaging features [1]. To date, no strategies have proved to be effective in screening and early diagnosis of ovarian cancer [2]. Numerous imaging modalities, including computed tomography (CT) and magnetic resonance imaging (MRI), have been proposed as useful adjuncts to conventional ultrasound for the detection of ovarian cancer. However, despite having higher sensitivity and specificity than ultrasound, preoperative diagnosis using CT or MRI is often indeterminate [3,4]. Until recently, most series have used ^18^F-fluorodeoxyglucose-positron emission tomography/CT (^18^F-FDG-PET/CT) to detect recurrence or distant metastasis [5-8]. In contrast, only a few studies have demonstrated the effectiveness of ^18^F-FDG-PET/CT in detecting primary ovarian cancer [9,10]. Despite high diagnostic value in identifying primary ovarian cancer, some pitfalls exist. False-positive results have been reported in patients with pelvic inflammatory disease (PID), tubo-ovarian abscess, and endometriosis [11,12], and false-negative results have been reported in patients with borderline tumors and early stage ovarian cancer [13].

To overcome this problem, acquisition of delayed images has been suggested. A previous study has demonstrated that ^18^F-FDG uptake continues to rise for several hours after the injection of the radiotracer in various malignancies [14]. This dual-time acquisition procedure has proved to be valuable in differentiating benign from malignant lesions in some solid tumors [15-19]. However, it has not been validated in patients with ovarian cancers.

The aim of our study is to establish the potential role of dual-phase ^18^F-FDG PET/CT for improving diagnostic accuracy of ovarian cancers.

## Methods

### Patients

From March 2009 to February 2011, we enrolled 20 consecutive patients presenting ovarian masses with diffuse peritoneal infiltration identified on abdomen–pelvis CT or pelvic MRI scans at Guro Hospital, Korea University. Each patient underwent conventional diagnostic work-up, including physical examination, pelvic ultrasound, and abdomen–pelvis CT or pelvic MRI. In all patients, images on CT or MRI suggested either benign or malignant masses with similar probability, making differential diagnosis difficult. To further confirm the characteristics of the masses, dual-phase ^18^F-FDG-PET/CT examinations were then performed in all patients. Patients who were pregnant, had a history of other types of cancers, or had liver cirrhosis, renal failure, or cardiovascular disease were excluded. All suspicious lesions were confirmed histopathologically by surgical exploration. The study protocol was approved by the Institutional Review Board for Research on Human Subjects at the Guro Hospital, Korea University, and written informed consent was obtained from all patients.

### Dual-phase ^18^F-FDG-PET/CT examination

All patients fasted for at least 6 h to maintain serum glucose level of <140 mg/dL before examination. One hour after intravenous injection of 555 MBq of ^18^F-FDG, each patient was scanned from the cerebellum to the pelvis using a PET/CT scanner (Gemini Time of Flight PET/CT, Philips, Amsterdam, the Netherlands). An additional delayed scan of the same area was taken 2 h after the ^18^F-FDG injection using the same scanner. Neither muscle relaxant nor contrast agent was administered.

### ^18^F-FDG-PET/CT data analysis

All images were interpreted and analyzed by 2 experienced nuclear medicine physicians with all available clinical information. PET images were analyzed on a dedicated workstation (Extended Brilliance Workspace 3.5 with PET/CT viewer for automated image registration, Philips, Amsterdam, the Netherlands). After image reconstruction, regions of interest were accurately placed over the lesion visualized on the PET images. All regions of interest were quantitatively evaluated using the standardized uptake value (SUV). The maximum SUV was calculated using the following formula:

$$\text{SUV}=\text{tissue}\;\text{concentration}\times \text{injected}\;{\text{dose}}^{‒1}\times \text{body}\;{\text{weight}}^{‒1}$$

For quantitative analysis of the uptake of the lesion, a region of interest was placed over the area of maximum activity within the lesion, and the maximum SUV (SUV_max_) was calculated from the measured tissue concentration at 1 h (SUV_max1_) and 2 h (SUV_max2_). The retention index (RI) was calculated by subtracting the SUV_max 1_from the SUV_max 2_and dividing by SUV_max 1_.

### Statistical analysis

The diagnostic accuracy of ^18^F-FDG-PET/CT in differentiating benign from malignant lesions was determined based on the histopathological findings. Numerical data such as age, parity, and body mass index were expressed as median (range). Mean percentage changes between SUV_max1_ and SUV_max2_ in both malignant and benign lesions were calculated, and were compared using the two-tailed Student’s *t*-test. Individual receiver-operating characteristic (ROC) curves of SUV_max1_, SUV_max2_, and RI were analyzed to determine their clinical significance in differentiating benign from malignant lesions. Pairwise comparisons of their performances were conducted using the Hanley and McNeil method. A *P* value of <0.05 was considered statistically significant. All statistical analyses were conducted using MedCalc Software for Windows (Version 11.6.0, MedCalc Software bvba, Ostend, Belgium).

## Results

Baseline characteristics of the enrolled patients are summarized in Table 1. Of the 20 patients, 13 were histopathologically diagnosed with primary epithelial ovarian cancers; 8 serous, 3 mucinous, and 2 endometrioid. The remaining 7 had non-cancerous lesions such as pelvic tuberculosis (3 patients), endometriosis (3 patients), and PID (1 patient). The median size of ovarian masses in 13 patients with primary epithelial ovarian cancers was 9.5 cm (range, 4–20), whereas the median size of ovarian masses in 7 patients with non-cancerous lesions was 3 cm (range, 1–7). In addition, all 20 patients showed diffuse peritoneal infiltration, such as peritoneal nodularity, omental or mesenteric infiltration, suggesting peritoneal carcinomatosis. Preoperative findings on CT or MRI scans in 13 patients with primary epithelial ovarian cancers mostly suggested malignancy as the first differential diagnosis, but other conditions could not be excluded using these scans, such as borderline ovarian malignancy, benign ovarian tumors, or soft tissue masses. In contrast, CT or MRI findings in 7 patients with benign lesions strongly suggested malignancies, but the possibilities of benign inflammatory lesions could not be completely excluded.

**Table 1:** Baseline characteristics of patients

**Characteristics**	**Malignant (*N * = 13)**	**Benign (*N * = 7)**
Age (years)	64 (50–80)	48 (30–69)
Parity	2 (0–3)	2 (0–4)
BMI (kg/m^2^)	23.5 ± 4.6	25.8 ± 5.5
Histopathology	8 serous	3 pelvic tuberculosis
	3 mucinous	3 endometriosis
	2 endometrioid	1 PID
Tumor size (cm)	7.5 (4–20)	3 (1–7)
SUV_max1_	7.7 (3.3-11.2)	3.2 (1.7-8.8)
SUV_max2_	10.1 (4.3-16.8)	4.3 (2.3-10.2)
RI	40 (16.2-50.0)	15.9 (1.2-35.3)

BMI = body mass index, SUV = standardized uptake value, RI = retention index, PID = pelvic inflammatory disease.

Data are presented as median (range) or mean ± SD.

**Figure 1:**
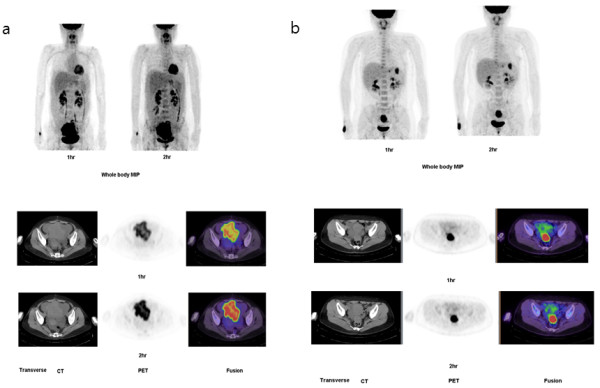
**The PET/CT images taken 1 h and 2 h after **^**18**^**F-FDG injection in a patient with ovarian cancer with peritoneal carcinomatosis (a) and pelvic tuberculosis (b). (a)** Whole body MIP image (upper). Axial image showed that FDG uptake at 2 h was significantly increased than FDG uptake at 1 h (lower) **(b)** Whole body MIP image (upper). Axial image showed almost no change of FDG uptake between at 2 h and at 1 h (lower). MIP = multiple intensity projection.

**Figure 2:**
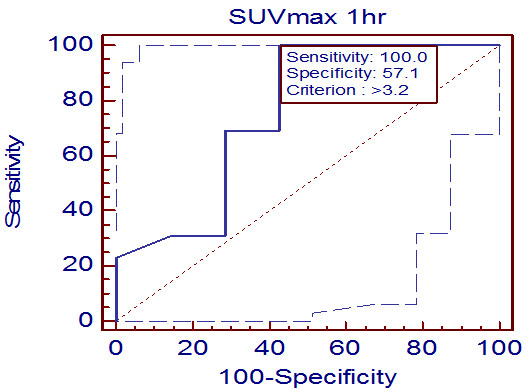
**Receiver operating curve analysis of standardized uptake value (SUV)**_**max1**_**.** The sensitivity and specificity were calculated when the SUV_max1_ cutoff value was set at 3.2.

**Figure 3:**
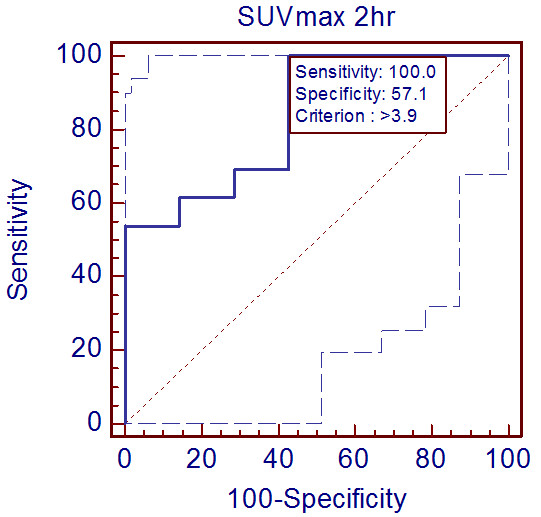
**Receiver operating curve analysis of standardized uptake value (SUV)**_**max2**_**.** The sensitivity and specificity were calculated when the SUV_max2_ cutoff value was set at 3.9.

**Figure 4:**
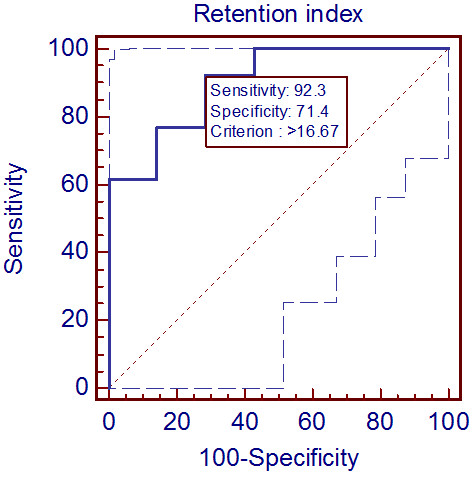
**Receiver operating curve analysis of retention index.** The sensitivity and specificity were calculated when the retention index cutoff value was set at 16.67.

The mean percentage changes between SUV_max1_ and SUV_max2_ in malignant and benign lesions were 2.6 ± 1.4 and 0.6 ± 0.4, respectively, indicating that SUV_max_ was significantly increased in malignant compared to benign lesions (*P* = 0.014). The PET/CT images taken 1 and 2 h after ^18^F-FDG injection in a patient with ovarian cancer and peritoneal carcinomatosis and in a patient with pelvic tuberculosis are presented in Figure 1. SUV_max1_ was 11.5 and 10.1 in a patient with ovarian cancer and a patient with pelvic tuberculosis, respectively. However, SUV_max2_ increased to 13.6 in the patient with ovarian cancer, whereas it decreased to 10.0 in the patient with pelvic tuberculosis. The results of the ROC analysis of SUV_max1_ are shown in Figure 2. The area under the curve (AUC) of SUV_max1_ was 0.753 (*P* = 0.063, 95% confidence interval [CI] = 0.512–0.915), indicating that was not a useful parameter in differentiating benign from malignant lesions. When the SUV_max1_ cutoff value was set at 3.2, the sensitivity and specificity were 100% and 57.1%, respectively. The results of the ROC analysis of SUV_max2_ are shown in Figure 3. In contrast, the AUC of SUV_max2_ was 0.835 (*P* = 0.001, 95% CI = 0.604–0.961), showing that the clinical usefulness of SUV_max2_ in differentiating benign from malignant lesions was statistically significant. When the SUV_max2_ cutoff value was set at 3.9, the sensitivity and specificity were 100% and 57.1%, respectively. Figure 4 shows the results of the ROC curve analysis of RI. The AUC was 0.901 (*P* < 0.001, 95% CI = 0.684–0.988), showing the greatest significance among the 3 parameters (SUV_max1_, SUV_max2_, and RI). When the RI cutoff value was set at 16.67, the sensitivity and specificity were 92.3% and 71.4%, respectively.

Pairwise comparison of the ROC curves was performed to compare the clinical utility of the 3 parameters in differentiating benign from malignant lesions. The AUC of SUV_max2_ was significantly higher than that of SUV_max1_ (*P* = 0.032). The AUC of RI was higher than both those of SUV_max1_ and SUV_max2_, but the differences obtained were not statistically significant.

## Discussion

The purpose of this study was to evaluate the value of dual-phase ^18^F-FDG-PET/CT in differentiating benign from malignant lesions in patients with indeterminate adnexal masses. We demonstrated that SUV_max2_ measured from images obtained from delayed phase scanning have a pivotal role when preoperative diagnosis is indeterminate. To the best of our knowledge, this is the first study to evaluate the clinical effectiveness of dual-phase ^18^F-FDG-PET/CT in the initial diagnosis of suspected malignant adnexal masses.

Although ^18^F-FDG-PET/CT is a useful tool in differentiating benign from malignant lesions, it cannot be used for specifically determining malignant lesions. Some infectious or inflammatory lesions can also cause high ^18^F-FDG uptake [20]. For example, Chen et al. [21] reported a patient with an adnexal mass referred for ^18^F-FDG-PET/CT scan owing to elevated tumor marker levels. The ^18^F-FDG-PET/CT scan showed multiple hypermetabolic foci in the peritoneum that mimicked peritoneal carcinomatosis. However, peritoneal tuberculosis was confirmed later. In our study, 3 out of 7 benign lesions were due to pelvic tuberculosis and mimicked peritoneal carcinomatosis. Conversely, most advanced stage ovarian cancers show peritoneal metastasis, and such peritoneal lesions often cause confusion during diagnosis because images obtained for these cancers are similar to those obtained for benign inflammatory conditions involving the gross peritoneal surface, as seen in our series.

Generally, whole-body ^18^F-FDG-PET/CT examination is performed 1 h after the administration of ^18^F-FDG. This seems to be ideal taking into account the half-life of the radiotracer, which is 110 min [17]. However, many different acquisition protocols have been proposed to overcome the aforementioned false-negative or false-positive results obtained with ^18^F-FDG-PET/CT. Saito et al. [15] used a protocol involving early-phase and delayed-phase scans (1 h and 2 h after ^18^F-FDG administration) in 48 consecutive patients with intraductal papillary mucinous neoplasia of the pancreas. In this study, SUV_max_ increased further in the delayed phase imaging in 92.3% patients with malignant pancreatic neoplasia, and only 60.0% of those with benign pancreatic neoplasia. This result is consistent with that of ours, in which SUV_max_ increased significantly in delayed phase PET/CT in malignant but not benign lesions. Cheng et al. performed dual-phase ^18^F-FDG-PET/CT in 35 patients with equivocal infiltrative hepatic lesions on abdominal CT or MRI scans [16]. In contrast to our study, the authors observed that SUV_max_ was significantly higher in infiltrative hepatic malignancies than in benign lesions in both early (5.9 ± 5.0 versus 3.9 ± 1.7, respectively) and delayed images (6.8 ± 10.2 versus 4.1 ± 3.9, respectively). Thus, early phase SUV_max_ of benign and malignant lesions were significantly different. In our study, only the AUC of SUV_max2_ was statistically significant in differentiating benign from malignant lesions. This might be attributed to either a different time point (45 min) for the early phase ^18^F-FDG-PET/CT examination or organ-specific characteristics. Caprio et al. evaluated the performance of dual-phase ^18^F-FDG-PET/CT in 48 patients suspected with breast lesions [17]. In their study, dual-time point acquisition of ^18^F-FDG-PET/CT displayed an accuracy of 85% for lesions with a SUV_max_ ≥ 2.5, with sensitivity and specificity values of 81% and 100% compared with 63% and 100%, respectively, for the single-time point acquisition. In our study, the sensitivity and specificity of the SUV_max2_ were 100% and 57.1%, respectively, with a SUV_max2_ > 3.9. The relatively small number of patients and the different time point of the (3 h) in delayed phase ^18^F-FDG-PET/CT examination might account for the differences in sensitivity and specificity. Yang et al. investigated whether adding delayed phase imaging can improve diagnostic ability of ^18^F-FDG-PET in evaluating solitary pulmonary nodules [18]. In their study, combined early and delayed phase scans of 28 patients showed correct diagnosis of the 3 malignant lesions with an initial SUV < 2.5. Despite minor differences in the study design and results compared to those of our study, the abovementioned studies confirmed the clinical usefulness of dual-phase PET/CT in differentiating benign from malignant lesions.

The major limitations of the current study include the small number of patients. Although patients were prospectively enrolled at the beginning of this study, selection bias could have been introduced. In other words, the clinicians’ attitudes to dual-phase PET/CT might affect the referral of patients. Second, this study was performed in a single institution. Differences in image acquisition and interpretation might influence the study results. Therefore, an additional large-scale, prospective study is necessary to check if our findings have an impact on future clinical practice. In addition, cost-effectiveness analysis should also be conducted. Despite these limitations, the findings of the present study provide useful information for the management of patients with equivocal adnexal masses; the sensitivities of both MRI and single-phase ^18^F-FDG-PET/CT for detecting ovarian cancer have reported to be similar [22,23]. Hence, if differential diagnosis of an adnexal mass is indeterminate by MRI, then probably the usefulness of single-phase ^18^F-FDG-PET/CT are also limited. Dual-phase ^18^F-FDG-PET/CT may be a better procedure in resolving this ambiguity. Accurate preoperative assessment could help clinicians perform prompt debulking surgery in patients with “true positive” ovarian cancers, and avoid unnecessary surgery in patients with benign conditions.

## Conclusion

SUV_max2_ obtained from dual-phase ^18^F-FDG-PET/CT is a useful parameter in differentiating benign from malignant lesions in patients with indeterminate adnexal masses. Further studies with larger patient populations are warranted.

## Abbreviations

FDG-PET/CT: Fluorodeoxyglucose positron emission tomography / computed tomography; SUV: The standardized uptake value; SUVmax: The maximum standardized uptake value; AUCs: The areas under the receiver operating characteristic curves; CI: Confidence interval; CT: Computed tomography; MRI: Magnetic resonance imaging; PID: Pelvic inflammatory disease; RI: Retention index.

## Competing interest

The authors declare that they have no competing interests.

## Authors’ contributions

SK and JHH devised the study and supervised the data collection. JKL and JHH performed wrote the manuscript. KAS and KJM contributed to the discussions and collected the data and performed the analyses. All authors contributed to the discussions. All authors read and approved the final manuscript.
